# Relative Risk of Acute Myocardial Infarction in People with Schizophrenia and Bipolar Disorder: A Population-Based Cohort Study

**DOI:** 10.1371/journal.pone.0134763

**Published:** 2015-08-13

**Authors:** Shu-I Wu, Su-Chiu Chen, Shen-Ing Liu, Fang-Ju Sun, Jimmy J. M. Juang, Hsin-Chien Lee, Kai-Liang Kao, Michael E. Dewey, Martin Prince, Robert Stewart

**Affiliations:** 1 Mackay Memorial Hospital, Department of Psychiatry, Taipei, Taiwan; 2 Mackay Medical College, Department of Audiology and Speech Language Pathology, Taipei, Taiwan; 3 National Taipei University of Nursing and Health Sciences, Department of Health Care Management, Taipei, Taiwan; 4 Mackay Junior College of Nursing, Taipei, Taiwan; 5 Mackay Memorial Hospital, Department of Medical Research, Taipei, Taiwan; 6 Cardiovascular Center and Division of Cardiology, Department of Internal Medicine, National Taiwan University Hospital and National Taiwan University College of Medicine, Taipei, Taiwan; 7 Department of Psychiatry, Shuang Ho Hospital, Taipei, Taiwan; 8 Department of Psychiatry, School of Medicine, College of Medicine, Taipei Medical University, Taipei, Taiwan; 9 Far Eastern Memorial Hospital, Department of Pediatrics, Taipei, Taiwan; 10 King’s College London, (Institute of Psychiatry, Psychology & Neuroscience), Health Service and Population Research Department, London, United Kingdom; Katholieke Universiteit Leuven, BELGIUM

## Abstract

**Objective:**

Despite high mortality associated with serious mental illness, risk of acute myocardial infarction (AMI) remains unclear, especially for patients with bipolar disorder. The main objective was to investigate the relative risk of AMI associated with schizophrenia and bipolar disorders in a national sample.

**Method:**

Using nationwide administrative data, an 11-year historic cohort study was assembled, comprised of cases aged 18 and above who had received a diagnosis of schizophrenia or bipolar disorder, compared to a random sample of all other adults excluding those with diagnoses of serious mental illness. Incident AMI as a primary diagnosis was ascertained. Hazard ratios stratified by age and gender were calculated and Cox regression models were used to adjust for other covariates.

**Results:**

A total of 70,225 people with schizophrenia or bipolar disorder and 207,592 people without serious mental illness were compared. Hazard ratios in men adjusted for age, income and urbanization were 1.15 (95% CI 1.01~1.32) for schizophrenia and 1.37 (1.08~1.73)for bipolar disorder, and in women, 1.85 (1.58~2.18) and 1.88(1.47~2.41) respectively. Further adjustment for treated hypertension, diabetes and hyperlipidaemia attenuated the hazard ratio for men with schizophrenia but not the other comparison groups. Hazard ratios were significantly stronger in women than men and were stronger in younger compared to older age groups for both disorders; however, gender modification was only significant in people with schizophrenia, and age modification only significant in people with bipolar disorder.

**Conclusions:**

In this large national sample, schizophrenia and bipolar disorder were associated with raised risk of AMI in women and in the younger age groups although showed differences in potential confounding and modifying factors.

## Introduction

People with serious mental illness experience significant functional decline and premature mortality [1]. Most research has focused on schizophrenia, which is associated with a 20% reduction in life expectancy compared with the general population[2–4]. Although people with serious mental illness have raised risk of suicide and other ‘unnatural’ mortality [5],standardized mortality ratios for ‘natural’ causes of death are also raised about 1.3- to 3-fold [4,6,7], and account for about 60%ofthe observed excess mortality in people with schizophrenia[5,6]. Several studies have suggested that this may be explained by a higher prevalence of physical comorbidities[1,3,7,8].The prevalence of cardiovascular disease(CVD) isparticularly high [9–12], and CVD has rapidly becomethe leading cause of death in people with serious mental illness [13,14]. Factors underlying these associations are likely to include socio-economic deprivation,and worse risk profiles including smoking, lack of exercise and obesity [15]. There is also a higher risk of disorders such as hypertension [9,16], diabetes [9,10,16], and dyslipidaemia [9,17,18]. Of potential relevance, these factors are alsoassociated with levels of urbanization (influenced by theincrease in the consumption of protein and fat, the decrease in energy expenditures[19]),and with psychotropic agents–especially antipsychotics, which may induce glucose or metabolic dysfunctions through direct molecular effects or increased abdominal adiposity[20–23].

Although previous research has investigated prevalent cardiovascular or coronary heart disease among people with schizophrenia, the risk of acute myocardial infarction (AMI) is less well documented. Those prospective studies which have sought to investigate this have resulted in heterogeneous findings with some reporting absent or negative associations between schizophrenia and AMI [10,24], while others have found increased incidence[9] or increased hazard shortly after the first episode[12].

In addition, most studies in the field have either defined a composite category of serious mental illness[11,25,26], or have reported on schizophrenia specifically [10,27]. Although mental disorder is well-recognized as associated with cardiovascular mortality [25], coronary heart disease has received less specific investigation as an outcome. On the other hand, less is known about differences in cardiovascular effects of common treatments given in bipolar disorder, such as mood stabilizers and antipsychotic agents [28]. From findings to date, the relative risk of mortality from ischemic heart disease or cardiovascular causes in people with bipolar disorder has ranged from 1.35 [8] to 1.67 [25]compared with controls, and higher incidence and prevalence of cardiovascular diseases have also been found [27,29]. One study found a higher but not significant odds ratio of 1.31 for risk of AMI in people with bipolar disorder aged 45 and above [30]; however, it is not clear whether this also applies to younger age groups.

Taking advantage of a large comprehensive national health insurance database available for research use in Taiwan, we sought to investigate the relative risk of AMI in cohorts of people with schizophrenia and bipolar disorder compared to residents without serious mental illness. Specifically we investigated modification by age and gender, and effect modification between the two disorders of interest.

## Methods

### Data source

The datasets used in this study were subsets from The TaiwanNational Health Insurance Research Database (NHIRD). The Taiwan National Health Insurance (NHI) program is a single- payer medical insurance system launched on March 1, 1995, which provides over 99% of all residents (about 22.8 million out of the total 23 million population) unrestricted access to all levels of health care (comparable to the NHS model in the UK)[31]. Anonymized information including records of drug prescriptions, use of medical services or interventions given, available from 1996 through the present, are collected, scrambled, and de-identified each year to form the original files of the NHIRD and are provided on request to scientists in Taiwan for research purposes[32]. The study described here was approved by the Mackay Memorial Hospital Institutional Review Board, protocol number 10MMHIS056.

### Study sample

From the NHIRD, subsets of the *Psychiatric Inpatients Medical Claim Dataset* (PIMC) and the *Longitudinal Health Insurance Research Database 2000* (LHID2000) [33]were used to define the case and comparison cohorts, respectively[31]. Both subsets contain records on all medical service use from 1996 to 2007. The PIMC was created in year 2000 and is formed by all (nearly 100,000) patients hospitalized in psychiatric departments from 1996 to 2000. The LHID2000 was also established in 2000 and is formed by 1 million individuals randomly selected from the 20 million total enrollees in the Taiwan NHI program. Detailed methods of random selection are described on the NHIRD website [34]. The random selection yielded no significant differences in the age or gender distributions between LHID2000, the NHIRD, and the known population distributions in Taiwan derived from census data [31,32].

#### Case cohort

The case sample was defined as people who were registered on either the PIMC and LHID2000 datasets, who were aged 18 or above at the time they received diagnosis of schizophrenia (ICD-9-CM 295.XX), or bipolar disorder (ICD-9-CM 296.XX, excluding major depressive disorder, ICD-9-CM 296.2X~296.3X)at any stage between 1996 and 2000. A hierarchical algorithm (based on the orders of organic disorder > schizophrenia> schizoaffective disorder or paranoia> bipolar disorder) was used for assigning psychiatric diagnoses in the case samples. Through this, a diagnosis of schizophrenia would be given if an individual had not received a diagnosis of organic mental disorder in the 11-year follow up period; similarly, an individual would be diagnosed with bipolar disorder only when diagnoses of schizophrenia, schizoaffective disorder, paranoia, or organic mental disorder had never been given in the 11-year period. People on the LHID2000 but not the PIMC who had been diagnosed with schizophrenia or bipolar disorder (i.e. as outpatients) between1996 and 2007 were also included, although this group was too small to analyze separately [31].

#### Control cohort

300,000 out of 1 million people registered on the LHID2000 subset were randomly selected (a simple random sample without stratification) as the comparison ‘control’ sample. Exclusion criteria for this group were under 18 years of age at the time of first medical visit and any diagnosis of organic mental disorder (ICD-9-CM 294.XX) or paranoid state (ICD-9-CM 297.XX) from 1996 to 2007. As mentioned previously, patients with diagnoses of schizophrenia or bipolar disorder from the LHID2000 or those who overlapped on both the PIMC and the LHID2000 datasets were re-classified as cases [31].

### Acute myocardial infarction ascertainment

The primary outcome of this study was defined as the first incident AMI (ICD-9-CM 410.XX) diagnosed from ambulatory care, emergency services, or hospitalization during 1996 to 2007. Evidence from electrocardiography and cardiac enzymes was required for this according to standard clinical practice in Taiwan. AMI occurrence prior to the surveillance period was not known; however, sensitivity analyses were carried out limiting the outcome to first AMI hospitalization in the latter half of the surveillance period.

### Covariates

The following measures available on the dataset were considered as covariates in this analysis: age at study entry, gender, level of urbanization in 5 strata (with level 1 representing the most urbanized, and level 5 the least urbanized)[12,31,35,36], monthly income in 4 strata[12], the presence of the following comorbid medical disorders in any records: hypertension, diabetes, hyperlipidaemia, and alcohol use disorder; as well as drug prescriptions including cardiovascular drugs (antihypertensive agents, antiplatelet agents, diuretics, nonsteroidal anti-inflammatory agents, antidiabetic agents, or lipid-lowering agents), antipsychotic agents, antidepressants, and mood stabilizers. In clinical practice under the NHI system in Taiwan, recording of appropriateICD-9-CM diagnoses is required in order for physicians to provide relevant interventions or prescriptions. Therefore, the presence of a coded medical disorder can be taken to imply treatment receipt.

### Statistical analysis

SAS version 9 for Windows (SAS Institute, Cary, NC) was used for data manipulation and assembly for analysis. The frequencies of target disorders were displayed. Pearson’s chi-squared tests were used for categorical variable comparisons and the Kruskal-Wallis test was used to investigate average differences in age at mental disorder diagnosis and/or AMI because this was not normally distributed in the three groups, but one-way ANOVA and the Bonferroni post hoc multiple comparison tests could still be performed due to central limit theorem. Corrections for multiple testing were also performed using Bonferroni *post hoc* multiple comparison tests. Colinearity between variables was checked prior to their inclusion in regression models. Proportional hazards (PH) assumptions were checked from Kaplan-Meier survival curves and by time-dependent covariate test [37]. Further Cox regression using stratifications by covariates that violated the PH assumptions were performed. Hazard ratios and 95% confidence intervals were obtained for the outcome stratified by gender. Cox models were used to estimate hazard ratios and to adjust other covariates for rate outcomes from the time of first psychiatric diagnosis between 1996 and 2007in the case group (i.e. cases who received their first mental disorder diagnosis after AMI were excluded), and from the time of first medical visit for the comparison group. Censoring points were the end of follow up or the date of withdrawal from the registry. For illustrative purposes, a figure stratified by age was constructed after effect modification for the associations of interest restricted *a priori* to age and gender was tested. In this respect, age group at study entry was entered as an ordinal variable on one degree of freedom, on the assumption that interactions of interest would show linear relationships with age. Sensitivity analyses using similar procedures restricted the sample to patients whose first AMI diagnosis occurred after 07.01.2001 (i.e. the latter half of the surveillance period) to check whether excluding earlier AMI (which might reflect recurrence rather than first episode) would cause meaningful changes to the results. We also carried out a sensitivity analysis using 01.01.1996 rather than date of first register entry as the starting date for comparison cohort; as well as a sensitivity analysis using a smaller sample containing only patients with or without serious mental illness from the total population cohort (LHID).

## Results

After applying inclusion/exclusion criteria there were 277,817 adults from the PIMC and LHID2000 datasets analyzed, of whom 58,106 and 12,119 had a diagnosis of schizophrenia and bipolar disorder respectively, and 207,592 were controls. Among the cases, 56,479 (97.2%) of those with schizophrenia, and 9,850 (81.3%) of those with bipolar disorder were contained on the PIMC register–i.e. had received their psychiatric diagnosis in the context of a hospitalization episode between 1996 and 2000. Of the 212,088 people on the LHIRD, 688 (31.5%) of all 2183 patients with schizophrenia were also on the PIMC (i.e. were cases that had received hospitalization), compared to 142 (6.1%) of all 2,313 patients with bipolar disorder.

Table 1 summarizes the characteristics of the cohorts. Significant differences were found in the average ages at study entry of three groups (Kruskal-Wallis *X* ^2^ 2697.84, df 2, p<0.001) and in all categorical variables (including age group) between people with and without serious mental illness (all p-values<0.001from chi-squared tests). Cases with schizophrenia had a higher male predominance and lower socio-economic status, than controls. On the other hand, people diagnosed with bipolar disorder had a higher female predominance, and higher proportions living in more urbanized areas compared to schizophrenia. Lower income level was found in those with mental disorders, particularly schizophrenia. Finally, people with the mental disorders were more likely to have the comorbid medical disorders (including hypertension, diabetes, hyperlipidaemia, and alcohol use disorders) and to have received cardiovascular prescriptions. Proportions receiving three months of antipsychotic use were 69.3% and 57.8% in schizophrenia and bipolar disorder respectively, and were 30.2% and 28.3% for six months of antipsychotic use[38–40]. Results from stratifying prescription patterns by mental illness and age groups showed that more antipsychotic agents and/or mood stabilizers were prescribed in the younger age groups (75.6% of patients with schizophrenia under age were receiving antipsychotic agents and 46.0% of those with bipolar disorder under age 45).

**Table 1:** Between-cohort comparison of demographic characteristics and comorbid medical disorders.

	No serious mental illness (n = 207,592)	Schizophrenia (n = 58,106)	Bipolar disorder (n = 12,119)	P value
Mean (SD) age at study entry	40.9 (16.3)	38.4 (13.1)	47.3 (16.4)	<0.0001
Age group at study entry (%)				<0.0001
18~34	46.4	50.3	29.1	
35~44	22.0	26.7	24.0	
45~54	12.0	12.4	17.6	
55~64	9.3	5.9	12.3	
65 and above	10.3	4.7	17.0	
Gender (%)				<0.0001
Men	52.5	56.9	45.3	
Women	47.5	43.1	54.7	
Gender missing (n)	102	0	2	
Levels of urbanization (%)				<0.0001
1 (most urbanized)	31.0	26.1	31.0	
2	30.3	30.3	32.9	
3	16.5	16.0	13.7	
4	13.2	14.6	13.8	
5 (least urbanized)	9.0	13.1	8.6	
Monthly income (%)				<0.0001
NT 0	19.4	17.1	24.2	
NT$ 1~15840	18.1	52.0	29.0	
NT$ 15841 ~ 25000	38.7	26.2	32.1	
≧NT$ 25001	23.8	4.7	14.7	
Hypertension (%)	24.0	25.8	29.5	<0.0001
Diabetes (%)	10.1	22.0	13.0	<0.0001
Hyperlipidaemia (%)	16.0	21.0	19.1	<0.0001
Alcohol use disorders (%)	0.8	15.7	11.6	<0.0001
Prescriptions				
Antipsychotic agents (%)	9.8	97.6	84.4	<0.0001
First Generation antipsychotic	9.5	95.1	82.5	
Second Generation antipsychotic	0.1	11.2	5.8	
Antidepressants (%)	5.7	66.6	77.1	<0.0001
Mood stabilizers (%)	1.8	52.9	58.9	<0.0001
Cardiovascular drugsa (%)	19.6	83.1	79.7	<0.0001
Aspirin	4.4	6.4	10.1	<0.0001
Statin	3.0	8.9	14.4	<0.0001
No exposures to antipsychotic or mood stabilizer(%)	89.2	2.3	14.6	<0.0001
Only exposed to antipsychotic agents(%)	8.9	44.7	26.2	<0.0001
Only exposed to mood stabilizers (%)	1.0	0.1	1.1	<0.0001
Exposed to both antipsychotic and mood stabilizers (%)	0.9	53.0	58.2	<0.0001

^a^ Cardiovascular drugs included antihypertensive agents, antiplatelet agents, diuretics, nonsteroidal anti-inflammatory agents, antidiabetic agents or lipid-lowering agents.

In the combined cohort of 277,817 people, 3,361 (1.21%) experienced at least one AMI episode during the 11-year follow up period: 591 (1.02%) in those with schizophrenia, 243 (2.00%) in those with bipolar disorder, and 2,527 (1.22%) in the comparison cohort. However, we need to take mean age of AMI into account. The mean (SD) age at recorded AMI was 57.1 (15.4) years in people with schizophrenia, 64.2 (15.4) in people with bipolar disorder, and66.8 (13.8) in the comparison cohort. Mean age of AMI was 9.7 (95% CI 8.1~11.2, p<0.0001) years lower in schizophrenia compared to controls, 2.6(95% CI 0.3~4.8, p<0.05) years lower in bipolar disorder compared to controls, and 7.1 (95% CI 4.5~9.7, p<0.0001) years lower in schizophrenia compared to bipolar disorder (p-values estimated using the Bonferroni post hoc multiple comparison test).

Kaplan-Meier survival curves and tests of time-dependent covariate showed that proportional hazard assumptions were not violated for most of covariates, except for hypertension, hyperlipidaemia, and cardiovascular drug use. Results from adjusted and further stratified Cox regression models, excluding 104 people with missing data on gender, and 228 people whose diagnosis of AMI was recorded prior to the diagnosis of mental disorder, are summarized in Table 2.Tests of modification by gender in fully adjusted models were significant for schizophrenia (hazard ratio for male gender x disorder interaction term:0.66,95% CI 0.54~0.81, p<0.001) but not for bipolar disorder (HR1.24,95% CI 0.90~1.72, p = 0.19). Addition of hypertension, diabetes, and hyperlipidaemia diagnoses to the models substantially attenuated the hazard ratios for schizophrenia in men but did not influence those for schizophrenia in women, or for bipolar disorder in either men or women. However, the hazard ratio in men for schizophrenia was attenuated after adjusting for cardiovascular drugs. Substantial attenuations in hazard ratios were also noted for both genders in both mental illnesses after adjustments of psychotropic agents (mood stabilizers, antipsychotics, and antidepressants).

**Table 2:** Hazard ratios (HR) of AMI in people with and without serious mental illness.^b^

		Model 1	Model 2	Model 3	Model 4	Model 5	Model 6
Schizophrenia	Total	**1.41 (1.28 ~ 1.57)**	**1.39 (1.25 ~ 1.54)**	**1.20 (1.08 ~ 1.33)**	1.07 (0.96 ~ 1.20)	0.95 (0.85 ~ 1.07)	**0.86 (0.74 ~ 0.99)**
	Male	**1.18 (1.03 ~ 1.35)**	**1.15 (1.01 ~ 1.32)**	0.98 (0.85 ~ 1.13)	**0.86 (0.74 ~ 1.00)**	**0.73 (0.63 ~ 0.86)** ^c^	**0.64 (0.54 ~ 0.77)**
	<50 years					**0.53 (0.42 ~ 0.67)**	
	>50 years					0.90 (0.74 ~ 1.10)	
	Female	**1.86 (1.59 ~ 2.19)**	**1.85 (1.58 ~ 2.18)**	**1.59 (1.35 ~ 1.88)**	**1.52 (1.28 ~ 1.80)**	**1.44 (1.20 ~ 1.72)**	1.15 (0.93 ~ 1.44)
Bipolar disorder	Total	**1.51 (1.28 ~ 1.79)**	**1.51 (1.27 ~ 1.79)**	**1.68 (1.42 ~ 2.00)** ^2^	**1.63 (1.37 ~ 1.94)**	**1.30 (1.09 ~ 1.55)**	1.15 (0.94 ~ 1.40)
	Male	**1.38 (1.10 ~ 1.74)**	**1.37 (1.08 ~ 1.73)**	**1.53 (1.21 ~ 1.94)**	**1.46 (1.15 ~ 1.85)**	1.10 (0.86 ~ 1.40)	0.96 (0.73 ~ 1.26)
	Female	**1.87 (1.46 ~ 2.40)**	**1.88 (1.47 ~ 2.41)**	**2.16 (1.68 ~ 2.78)**	**2.14 (1.67 ~ 2.76)**	**2.02 (1.56 ~ 2.60)**	**1.49 (1.11 ~ 1.99)**

^b^104 people with gender missing, 131 people with schizophrenia and 97 people with bipolar disorder who had AMI incident prior to mental disorder diagnosis were not included in the analysis. Stratified analyses were performed for non-proportional hazards covariates (hypertension, hyperlipidemia, and/or cardiovascular drug use).

^c^Non-propotional hazards, one should mainly look at its results of gender or age strata (further stratification by gender in Model 3 for bipolar disorder and by younger or older than age 50 in Model 5 for male patients with schizophrenia were performed due to findings from time-dependent covariate test).

Model 1: adjusted for age at study entry. Model 2: adjusted for gender (where not stratified), age at study entry, levels of income and urbanization. Model 3: adjusted for gender (where not stratified), age at study entry, levels of income, levels of urbanization, hypertension, diabetes, and hyperlipidaemia. Model 4: adjusted for gender (where not stratified), age at study entry, levels of income and urbanization, hypertension, diabetes, hyperlipidaemia, and alcohol use disorder. Model 5: adjusted for gender (where not stratified), age at study entry, levels of income and urbanization, hypertension, diabetes, hyperlipidaemia, and cardiovascular drug use. Model 6: adjusted for gender (where not stratified), age at study entry, levels of income and urbanization, hypertension, diabetes, hyperlipidaemia, and psychotropic use.

**Fig 1:**
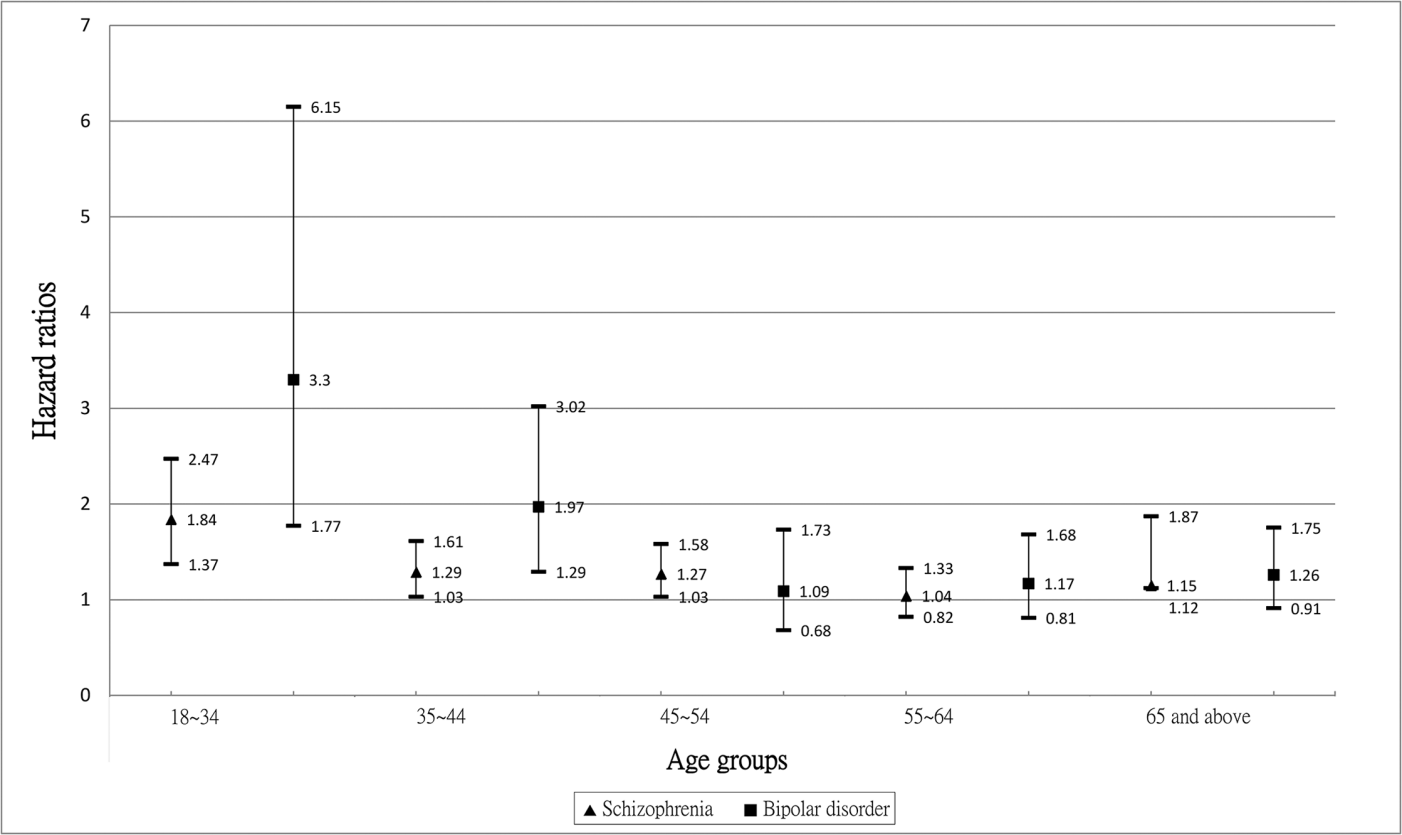
Age-stratified hazard ratios and 95% confidence intervals of AMI in people with or without schizophrenia or bipolar disorder (adjusted for age at study entry, levels of income and urbanization). ▲ Schizophrenia ∎ Bipolar disorder.

The interaction term with age was significant for bipolar disorder (fully adjusted HR 0.98, 95% CI 0.97~0.99, p = 0.005) indicating a stronger excess risk in younger people, but this was not significant for schizophrenia (fully adjusted HR 0.99, 95% CI: 0.99~1.00, p = 0.171). For illustrative purposes, Fig 1 summarizes age stratified models for schizophrenia and bipolar disorder. It was found that risks of AMI in the two mental disorder cohorts were raised within almost all age groups for men and women with bipolar disorder, and for women with schizophrenia, but these were more equivocal for men with schizophrenia. Three-way (age group x gender x disorder) interaction terms were tested in fully adjusted models but were not found to be significant for either disorder (p-values >0.10).

Results from sensitivity analyses restricted to AMI ascertained in the latter half of the surveillance period were, in essence, identical to those obtained in the analyses of the full sample (data not shown). The sensitivity analysis using 01.01.1996 rather than date of first register entry as the starting date for comparison cohort again made no meaningful difference to the findings. Results from the sensitivity analysis utilizing a smaller sample containing only patients with serious mental illness from the total population cohort (LHID) revealed a significantly stronger hazard ratio in men with bipolar disorder in addition to findings obtained from the full sample, though only gender x disorder interaction was observed in people with bipolar disorder.

## Discussion

In this large analysis of national health care records, people with schizophrenia and bipolar disorder were found to have a significantly increased risk of AMI over an 11-year surveillance period compared to people without serious mental illness. The mean age of AMI was also substantially lower in both case cohorts than in the comparison groups. Gender modification was observed in people with schizophrenia, with risk associations particularly strong in women. Age modification was observed, with younger group in people with bipolar disorder showing stronger risk of AMI.

Our investigation of the risk of AMI in people with mental illness was carried out on what we believe is the largest database of psychiatric inpatients to date, comparing these with a nationally representative sample of controls. Our finding is supported by previous research on prevalence and short term incidence [9,12], and is in line with other publications describing higher risks of cardiovascular diseases among people with schizophrenia [11,41,42]. As outlined earlier, there are a variety of potential causal pathways between serious mental disorders and cardiovascular outcomes, including adverse conventional risk profiles. Of interest, adjustment for treated hypertension, diabetes, and hyperlipidaemia substantially attenuated the association between schizophrenia and AMI in men but made relatively little impact on this association in women or on associations with bipolar disorder in both men and women. This suggests that causal pathways may not be homogeneous in all groups or for both disorders. Although some studies have suggested that people with schizophrenia may live with an unhealthy lifestyle from an early age, with predisposition to certain disorders [43] (including higher susceptibility to poor glycaemic control and diabetes [44], higher levels of cholesterol, triglycerides and lower levels of high-density lipoprotein cholesterol[45], and/or abnormalities in the immune system[46] before any initiation of psychotropic medication, it could be that those male schizophrenia patients (especially those younger than 50 years of age, as seen from KM curve) with hypertension and/or hyperlipidaemia being identified and treated with cardiovascular drugs have received better supervisions on physical health while remaining in contact with mental health services compared to male of same age from general population. Intriguingly, we also found from Cox regression for Model 5 after age stratification (in order to conform to the proportional hazard assumption) that higher income level seemed to serve as a protective factor in male schizophrenia patients under age 50 (HR 0.86, 95% CI: 0.77~0.96, p = 0.007) but not in those above age 50. Therefore effects of differences in income levels might be more prominent in the younger age group of male schizophrenia patients than in the older ones. Finally, it is also possible that perhaps men with psychiatric conditions are at the highest of adverse cardiac prognosis that they were more likely to die and were therefore unable to remain in our dataset. Since we did not have access to the National Death Registry, further research on exploring mortality and the risk of interest among male patients with schizophrenia is still warranted. On the other hand, schizophrenia is a chronic disorder, and up to 50% of patients receiving long-term antipsychotic treatment [39] develop weight gain or metabolic syndrome [20,22,47–49]. Although in our study, about one-third of people with serious mental illness received antipsychotic treatment for over six months, the attenuation in the association of interest after adjusting for psychotropic agents (Model 6) might still imply a possible role of these medications in elevating the risk of AMI. Therefore, as the PRIMROSE study has suggested [18], exposures to antipsychotic agents, antidepressants, or mood stabilizers should be considered in the risk prediction and prevention of cardiovascular diseases among people with serious mental illness.

Much less research has investigated the risk of AMI in people with bipolar disorder. Previous studies have examined such associations either cross-sectionally [29], or longitudinally but with smaller numbers of bipolar patients [30,41], and none to date have compared AMI risk and its correlates between bipolar disorder and schizophrenia. Our findings indicate broadly similar adjusted hazard ratios for schizophrenia and bipolar disorder as exposures in women and similar hazard ratios in men prior to adjustment for treatment of vascular risk factors. Of note, regarding potential differences in causal pathways between the two disorders, it has been suggested that behavioral or adverse physiological changes (such as increased platelet aggregation and decreased heart rate variability) during depressive or manic episodes [50,51] might mediate at least some of the association with increased risk of cardiovascular diseases. On the other hand, although the two disorders might have high heritability in relatedness, there are also differences between them, including genetic variations related to smoking in people with schizophrenia but not bipolar disorder [52], or genetic differences leading to dysregulations in immune responses[53], which might lead to different cardiovascular effects. With schizophrenia having an earlier peak age of onset than bipolar disorder, and/or potentially a more insidious onset, patients with schizophrenia might suffer from longer periods of developmental disruptions, occupational function decline, social withdrawal, inactivity, or negativism, which might also cause differences in cardiovascular risk profiles between schizophrenia and bipolar disorder. Finally, although we were unable to disentangle the specific contributions of first or second generation antipsychotic agents and mood stabilizers, attenuation in the risk of AMI after adjusting for psychotropic use (Model 6) in both schizophrenia and bipolar disorder, might indicate a raised risk of AMI through possible metabolic changes or arrhythmogenic effects.

We found that women with schizophrenia or bipolar disorder had a 1.5-fold higher adjusted risk of AMI than comparison groups, which is supported by recent literature [9]. More specifically, in contrast to the recognised higher risk of coronary artery disease in young men compared with premenopausal women observed in most community populations, the cardioprotective effect in younger women wasapparently attenuated in the mental disorder groups. A large component of the reduced risk of coronary artery disease in premenopausal women is thought to be derived from a relatively favourable lipid profile, with higher levels of high-density lipoprotein cholesterol [54]. It is therefore important to establish mechanisms underlying the apparent negation of this protective effect [9], perhaps through metabolic syndrome, smoking, lack of exercise, or decreased levels of estradiol as suggested by previous research [9,55,56]. Of interest,this gender difference was more marked in schizophrenia compared to bipolar disorderand may not therefore be a generic effect of having a mental disorder.

Risks of AMI were raised nearly two-fold in younger people with schizophrenia (age under 35) and bipolar disorder (age under 45). In addition, age at AMI was approximately ten years and three years younger in the schizophrenia and bipolar groups respectively compared to the control group. Not all studies have found elevated risk of AMI associated with serious mental illness, and our results conflict from a prior report of absent or negative associations with AMI in people with bipolar disorder [30]. An important reason underlying this might be the age modification observed in our sample, especially as the other study focused on middle aged or older populations [30], in whom associations were absent or negative in our cohorts. The finding that more antipsychotic agents and/or mood stabilizers were prescribed in the younger age groups may be one of the reasons why the younger age groups were particularly vulnerable to the outcome of interest. The risk of AMI in younger age groups of patients with serious mental illnesses has generally been found to be associated with conventional risk factors such as impaired glucose tolerance, high triglyceride or low high-density lipoprotein levels and central obesity [57]. However, different risk profiles have also been suggested in the pathophysiology of very young AMI including congenital coronary abnormalities, hypercoagulability, illicit drugs causing vasospasm, and vasculitis resulting in coronary aneurysm and/or dissection [57].These and other more disorder-specific factors should also therefore be considered in populations with bipolar disorder [58], given the marked age modification observed. For example, a finding from the Coronary Artery Risk Development in Young Adults (CARDIA) study was that young people with an above median score on a hostility index had 2.6 times higher odds of coronary calcification [58],a finding which may have particular relevance to people with bipolar disorder during manic phases, or in young patients with schizophrenia during acute psychosis. It is also possible that different psychotropic medication agents or regimes were administered to younger patients. The relatively lower AMI risk in older people with serious mental illnesses may be accounted for by healthy survivor effects (and, of note, hypertension, hyperlipidaemia, and diabetes diagnoses were also relatively low in these groups(data not shown)), or as discussed earlier, because people appearing in the cohort have remained in contact with mental health services (i.e. potentially have higher treatment adherence and/or more attention paid by supervising clinicians to physical health and lifestyle).

Major strengths of this study include the prospective database large enough to provide sufficient statistical power for detecting the associations of interest, as well as its national representativeness and derivation from a healthcare system that covers all medical services, interventions, and drug prescriptions of its residents [31]. A key limitation is that findings were drawn from administrative rather than research datasets. In particular, diagnoses were clinician-initiated which do not necessarily generalize to research diagnostic criteria, although for this reason we deliberately chose case categories which were relatively broad and unambiguous, and there are compensating advantages in generalisability to naturalistic clinical environments. In terms of exposure, it is important to bear in mind that the cases predominantly represented relatively severely affected subgroups of people with schizophrenia or bipolar disorder since the majority had been hospitalized and there were insufficient cases drawn from the LHID2000 dataset alone (i.e. those without hospitalization) to analyze separately. This issue is particularly pertinent for bipolar disorder where a smaller proportion of LHID2000 cases appeared on the PIMC dataset compared to schizophrenia. A further limitation is that there was no information on lifetime history of mental disorders and we were only able to ascertain caseness on the basis of medical contact during the follow-up window. However, measurement errors in both exposure and outcome ascertainments will have obscured rather than exaggerated the associations of interest. It is important to bear in mind that the outcome did not include sudden cardiac deaths outside hospital or instances of AMI or other causes of mortality which did not result in hospitalization, because we did not have access to the National Death Registry; however, it would include instances of AMI resulting in early inpatient mortality if sufficient prior investigations had been carried out to ascertain AMI as the cause of this. Finally, although we were able to adjust for demographic status and certainmedical disorders, information was not available on other determinants of vascular risk status such as smoking or exercise habits, blood pressure levels, obesity or body size. These are therefore left as potential causal pathways between exposure and outcomeon which we are not able to comment.

## Conclusions

Using a nationwide database, we found a raised risk of AMI in people with schizophrenia and bipolar disorder but could only partially explain these raised risks by demographic characteristics and medical comorbidities. Our findings suggest a very worrying pronounced increase in AMI risk associated with both mental disorders in women and in younger adults, and future investigations need to focus on clarifying causal pathways, particularly focusing on women and younger people with serious mental illness. Given the recognised increased risk of cardiovascular mortality and substantially reduced life expectancy in people with major mental disorders, research is also required into how much this is accounted for by AMI risk and incidence and how much by subsequent interventions received by people with mental disorders who experience an AMI. Our findings have important potential clinical and policy implications concerning attention paid to physical healthcare in people with chronic severe mental disorders. These include a need for more rigorous medical attention to cardiovascular risk profiles, applying relevant cardiovascular risk prediction models as described in the PRIMROSE study[18] for early detection, and providing educational programs to raise awareness of healthy lifestyles in those affected and their caregivers.
